# TRP Channels: Current Perspectives in the Adverse Cardiac Remodeling

**DOI:** 10.3389/fphys.2019.00159

**Published:** 2019-03-01

**Authors:** Debora Falcón, Isabel Galeano-Otero, Eva Calderón-Sánchez, Raquel Del Toro, Marta Martín-Bórnez, Juan A. Rosado, Abdelkrim Hmadcha, Tarik Smani

**Affiliations:** ^1^ Department of Medical Physiology and Biophysics, Institute of Biomedicine of Seville, University of Seville, Sevilla, Spain; ^2^ CIBERCV, Madrid, Spain; ^3^ Department of Physiology (Cell Physiology Research Group), University of Extremadura, Cáceres, Spain; ^4^ Department of Generation and Cell Therapy, Andalusian Center for Molecular Biology and Regenerative Medicine (CABIMER), University of Pablo de Olavide-University of Seville-CSIC, Sevilla, Spain; ^5^ CIBERDEM, Madrid, Spain

**Keywords:** calcium, TRP channels, cardiac remodeling, hypertrophy, fibrosis, conduction disorders

## Abstract

Calcium is an important second messenger required not only for the excitation-contraction coupling of the heart but also critical for the activation of cell signaling pathways involved in the adverse cardiac remodeling and consequently for the heart failure. Sustained neurohumoral activation, pressure-overload, or myocardial injury can cause pathologic hypertrophic growth of the heart followed by interstitial fibrosis. The consequent heart’s structural and molecular adaptation might elevate the risk of developing heart failure and malignant arrhythmia. Compelling evidences have demonstrated that Ca^2+^ entry through TRP channels might play pivotal roles in cardiac function and pathology. TRP proteins are classified into six subfamilies: TRPC (canonical), TRPV (vanilloid), TRPM (melastatin), TRPA (ankyrin), TRPML (mucolipin), and TRPP (polycystin), which are activated by numerous physical and/or chemical stimuli. TRP channels participate to the handling of the intracellular Ca^2+^ concentration in cardiac myocytes and are mediators of different cardiovascular alterations. This review provides an overview of the current knowledge of TRP proteins implication in the pathologic process of some frequent cardiac diseases associated with the adverse cardiac remodeling such as cardiac hypertrophy, fibrosis, and conduction alteration.

## Introduction

Calcium (Ca^2+^) is an important second messenger necessary for the excitation-contraction (EC) coupling process in cardiac myocytes (Berridge, 2002; Bers, 2002; Eisner et al., 2017), which occurs as a consequence of Ca^2+^ entry to the cytosol due to L-type Ca^2+^ channels that provoke Ca^2+^ release from sarcoplasmic reticulum causing cardiac myocyte contraction. Cardiac relaxation starts when intracellular Ca^2+^ concentration ([Ca^2+^]_i_) decreases as a result of the activity of sarco/endoplasmic reticulum Ca^2+^ ATPase (SERCA) responsible for the Ca^2+^ reuptake into the sarcoplasmic reticulum and the Na^+^/Ca^2+^ exchanger responsible of the Ca^2+^ extrusion out of cardiomyocytes (Bers, 2002; Eder, 2017). Ca^2+^ is also required for the activation of signaling pathway that plays minor roles in the healthy heart, for example, those involved in the cardiac remodeling and heart failure (Yue et al., 2015; Eder, 2017; Freichel et al., 2017; Avila-Medina et al., 2018; Domínguez-Rodríguez et al., 2018).

**Figure 1:**
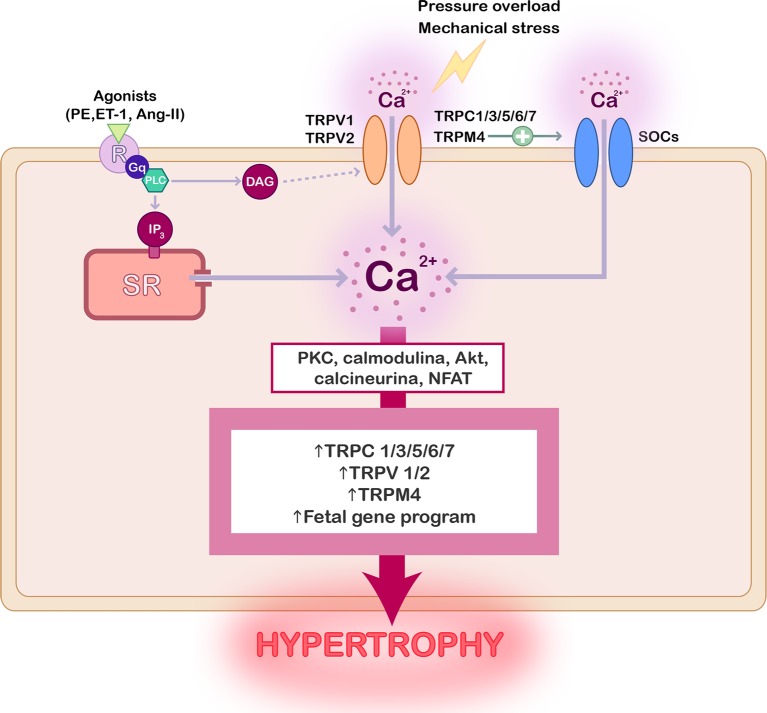
Scheme summarizing the role of TRP channels in cardiac hypertrophy. Activation of TRP channels can be preceded by stimulation of G-coupled receptors with hypertrophic agonists, by mechanical stress, or pressure overload. The consequent increase of the intracellular Ca^2+^ concentration stimulates different signaling protein, such as PKC, AKT, calcineurin, and NFAT, whose activation promotes TRP channels overexpression and the activation of fetal genes reprograming leading to cardiac hypertrophy.

Among the wide Ca^2+^-permeable channels known in the heart, transient receptor potential (TRP) channels contribute to the Ca^2+^ influx induced by a wide spectrum of physico-chemical stimuli from cellular microenvironments, such as thermal, mechanical stresses, and neurohormonal (Freichel et al., 2017). Likewise, a wide variety of vasoactive agent, including endothelin-1, thrombin, ATP, angiotensin-II, or bradykinin, also stimulates TRP (Suzuki et al., 2011; Albarrán et al., 2013; Gerhold and Schwartz, 2016; Sawamura et al., 2017). TRP channels comprise a large Ca^2+^-permeable cation channel superfamily showing a common architecture. They contain six transmembrane domains (TM1–TM6) and the cation-permeable pore region formed by a loop between TM5 and TM6 (Gaudet, 2007). They are divided into six major subgroups based on their specific function and sequence analogies (Nilius and Droogmans, 2001; Owsianik et al., 2006; Peng et al., 2015): the canonical channel (TRPC), the vanilloid-related channel (TRPV), and the melastatin-related channel (TRPM) formed by 7, 6, and 8 different channel proteins, respectively (Montell et al., 2002; Venkatachalam and Montell, 2007; Yu et al., 2010); the ankyrin-related channel (TRPA) subfamily containing only one protein; the mucolipin-related channel (TRPML) formed by three proteins; and polycystin-related channel (TRPP) constituted by three members also known as polycystic kidney disease protein (PKD2) (Montell et al., 2002; Venkatachalam and Montell, 2007).

TRP channels display diverse cation selectivity and activation mechanisms (Venkatachalam and Montell, 2007). Most of them are nonselective for cation and are permeable to monovalent and divalent cations, more permeability for Ca^2+^ than Na^+^ (ratio P_Ca_/P_Na_) that ranges from channels selective for monovalent cations, such as TRPM4 and TRPM5 with a ratio P_Ca_/P_Na_ < 0.05, to highly Ca^2+^ selective channels, as TRPV5 and TRPV6, which exhibit a ratio P_Ca_/P_Na_ close to 100 (Yue et al., 2015). TRPM6 and TRPM7 are also permeable to Mg^2+^, Ca^2+^, Na^+^, Zn^2+^, and other metals [for an extensive review see Freichel et al. (2017)]. Most of TRP channels lack the typical voltage sensor; therefore, they are not gated by voltage (Ramsey et al., 2006; Alonso-Carbajo et al., 2017; Jardín et al., 2017) but they can be modulated by a different chemical and physical stimuli, including temperature fluctuations and mechanical stretch (Islas, 2017; Nazıroğlu and Braidy, 2017; Yamaguchi et al., 2017), extracellular and intracellular ions (including H^+^, Ca^2+^, and Mg^2+^) (Launay et al., 2002; Jiang et al., 2005; Li et al., 2007), intracellular ligands [as diacylglycerol (Palazzo et al., 2013), phosphoinositide-4,5-bisphosphate (PIP2) (Ong and Ambudkar, 2017)], and various exogenous natural and synthetic ligands (Vetter and Lewis, 2011; Holzer and Izzo, 2014).

TRP channels have been suggested as regulators of local micro domains signaling pathway related with changes in the [Ca^2+^]_i_ or by the interplay with Ca^2+^-dependent regulatory proteins. Actually, they contribute to Ca^2+^ homeostasis by directly conducting Ca^2+^ or indirectly *via* membrane depolarization and modulation of voltage-gated Ca^2+^ channels (Nilius and Droogmans, 2001; Owsianik et al., 2006; Freichel et al., 2014; Pires et al., 2017). Hence, during the last two decades, TRPs have been suggested as intermediaries of diverse physiological and pathophysiological cardiovascular processes (Inoue et al., 2006, 2018; Egginton, 2009; Smani et al., 2015; Yue et al., 2015).

## Expression of TRP Channels in Cardiac Cells

RT-PCR, western blot, immunostaining, and functional current recordings demonstrated that TRPs are expressed ubiquitously in cardiac myocytes and fibroblasts of different species (Sabourin et al., 2011; Yue et al., 2015). In the case of TRPC channel, the seven members TRPC1–7 are expressed in the majority of the cell types in heart (Eder, 2017; Freichel et al., 2017). Consistently, all TRPCs, except TRPC5, were detected in the sinoatrial node (Ju et al., 2007). Interestingly, significant overexpression of TRPC1/C3/C4/C5 or TRPC6 was detected in patients with heart failure as compared to nonfailing heart (Bush et al., 2006; Morine et al., 2016). Interestingly, these TRPC channels show distinct profiles of expression in the ventricles of patients with heart failure as it happens in murine models of univentricular pressure overload (Morine et al., 2016). As in other cell types, TRPC channels are implicated in signal transduction in cardiac myocytes (Flockerzi and Nilius, 2014; Eder, 2017; Freichel et al., 2017). TRPC family requires the phospholipase C (PLC) pathway for activation. TRPC3, TRPC6, and TRPC7 interact directly with diacylglycerol (Yamaguchi et al., 2018), while TRPC1, TRPC4, and TRPC5 are activated indirectly through a still unidentified mechanism (Sabourin et al., 2011; Zhang and Trebak, 2014; He et al., 2017). Some TRPC channels are activated by intracellular Ca^2+^ store depletion, which stimulates the store-operated Ca^2+^ entry (SOCE) required for diverse cardiac physiopathological process (Ong et al., 2016; Eder, 2017). It has been proposed that TRPC1 associates with TRPC4 or TRPC5, thereby forming the store-operated Ca^2+^ channel, while TRPC3, TRPC6, and TRP7 are suggested to form the receptor-operated channel (Ju and Allen, 2007; Saleh et al., 2008; Sabourin et al., 2012). Others studies demonstrated that long-term stimulation of cardiac myocytes with angiotensin II, phenylephrine, endothelin-1, or aldosterone evoked an exacerbated SOCE elicited by thapsigargin, correlating with an increment in the expression or activation of TRPC1, TRPC4, and/or TRPC5 (Watanabe et al., 2008; Makarewich et al., 2014; Camacho Londoño et al., 2015; Sabourin et al., 2016). The use of dominant negative mutants confirmed that TRPC4 is sensitive to passive Ca^2+^ store depletion, while TRPC3 and TRPC6 respond to the diacylglycerol stimulus, regardless of store depletion (Makarewich et al., 2014). Furthermore, upregulation of TRPC3/C4 in adult ventricular cardiomyocytes correlated with the enhanced SOCE and pro-arrhythmic spontaneous Ca^2+^ waves (Domínguez-Rodríguez et al., 2015). Importantly, transient occlusion of coronary artery in rats also enhanced the expression of TRPC1/C3/C4/C5 and TRPC6 either in risk or in remote zone of the infarcted heart (Domínguez-Rodríguez et al., 2018). Finally, TRPC7 activation was proposed to initiate angiotensin-II activation to myocardial apoptosis (Satoh et al., 2007).

TRPV channels were also detected in mammalian hearts, especially TRPV1, TRPV2, and TRPV4 (Yue et al., 2015). Most of TRPV channels are sensitive to temperature and ligands, and they participate in sensation of hot temperature and in chemoreception (Vriens et al., 2007; Islas, 2017). TRPV1 was identified principally in sensory nerves in the cardiovascular system but also in the myocardium (Zahner et al., 2003; Gao et al., 2015; Randhawa and Jaggi, 2017). Bradykinin evoked a TRPV1-dependent [Ca^2+^]_i_ increase in cardiac neurons, indicating that TRPV1 activation was responsible for stimulation/sensitization by bradykinin of cardiac nociceptors (Wu and Pan, 2007). An early study demonstrated that after *trpv1* gene deletion, an exacerbated inflammation and cardiac remodeling occurred due to impaired post-ischemic recovery in isolated perfused infarcted heart (Wang and Wang, 2005). More recently, the overexpression of TRPV2 after myocardial infarction was observed in cardiac tissue of rats (Entin-Meer et al., 2014), and TRPV2 downregulation in knockout mice was related to a better recovery after myocardial infarction (Entin-Meer et al., 2017), probably because of an attenuated pro-inflammatory response in these mice. Another study also suggested that TRPV2 may play a critical role in stretch-activated Ca^2+^ influx pathway in dystrophic cardiomyopathy, contributing to [Ca^2+^]_i_ mishandling (Lorin et al., 2015). In the case of TRPV4, it is also highly expressed in the heart and is activated during myocardial ischemia and reperfusion, which induced Ca^2+^ influx with subsequent reactive oxygen species (ROS) release (Wu et al., 2017). Recently, TRPV4 upregulation in cardiomyocytes was also linked with aging in mice (Jones et al., 2018). Indeed, pharmacological inhibition of TRPV4 with HC067047 prevented stress-induced cardiomyocyte death and ischemia and reperfusion-induced cardiac damage in aged mice. These findings might have potential implications in the treatment of elderly populations at increased risk of myocardial infarction.

Regarding the expression of TRPM channels in hearts, it is known that eight isoforms are present in different parts of the heart, and TRPM2/4/7 is expressed specifically in cardiac myocytes (Yue et al., 2015). The analysis of mRNA and/or protein levels showed that levels of TRPM2, TRPM3 and TRPM8 were reduced in left and right ventricle of patients with failing heart (Morine et al., 2016), meanwhile increased expression of TRPM7 was observed in the left ventricle of patients with ventricular tachycardia (Parajuli et al., 2015). TRPM2 seems essential for cardiac myocyte bioenergetics maintenance (Hoffman et al., 2015). Its activation protected the heart from ischemia and reperfusion injury by improving mitochondrial dysfunction and reducing ROS levels (Miller et al., 2014). TRPM4 is thought to interfere in cardiac myocyte contraction by its activation of voltage-gated Ca^2+^ channel (Alonso-Carbajo et al., 2017). Actually, the exacerbated activity of TRPM4, which is activated by physiological range of Ca^2+^ concentration, was related with arrhythmic changes (Hu et al., 2017). Also, TRPM7 is believed indispensable during the myocardial proliferation in early stages of cardiogenesis, since the deletion of *trpm7* gene, before embryonic day 9 of mice, provoked heart failure and embryonic death (Sah et al., 2013). Nevertheless, the mechanism by which TRPM7 regulates cardiac cell proliferation remains unknown (Chubanov et al., 2017).

In relation to the expression and function of other TRP channels in heart, TRPA1 was detected in cardiac endothelial cells, vascular smooth muscle, and in cardiac myocytes (Yue et al., 2015), where its activation seems relevant to attenuate ischemia and reperfusion injury (Lu et al., 2016). Furthermore, the presence of TRPP2 has been proven in knockout mice who died before birth as a result of cardiac malformations (Pennekamp et al., 2002). Recently, a study confirmed that TRPP2 was able to regulate autophagy through Ca^2+^ homeostasis in cardiac myocytes (Criollo et al., 2018). However, there are only few studies which looked on the role of these channels in the function of cardiac cells.

## Role of TRP Channels in the Adverse Cardiac Remodeling

Ca^2+^ plays critical role in the adaptation of the heart to environmental demands, leading to cardiac remodeling (Eder, 2017; Avila-Medina et al., 2018). Physical exercise or pregnancy are reversible stimuli that induce physiologic cardiac hypertrophy to adapt the increase in consumption of nutrients and oxygen, whereas sustained neurohumoral activation, hypertension, or myocardial injury can lead to pathological heart hypertrophy followed by interstitial fibrosis (Pfeffer and Braunwald, 1990; Klug et al., 1993; Hill and Olson, 2008). These events might cause left ventricular dilation and dysfunction, what is known as the adverse cardiac remodeling, which increases the risk of heart failure and malignant arrhythmia (Hill and Olson, 2008; van der Bruggen et al., 2017).

Initial attentions have been given to describe the role of TRP channels in the appearance of cardiac remodeling using different experimental procedures *in vitro* and *in vivo* in animal models (Guinamard and Bois, 2007; Eder and Molkentin, 2011; Yue et al., 2015). Here, we will focus on the role of TRPCs, TRPVs, and TRPMs role in the adverse cardiac remodeling, since little is known regarding the other subfamilies and their participation in these processes.

### TRPs and Cardiac Hypertrophy

Compelling evidence confirmed that the activity and expression of several TRP channels are upregulated in pathological hypertrophy and heart failure as reviewed elsewhere (Watanabe et al., 2008; Yue et al., 2015; Freichel et al., 2017), and as summarized in Figure 1. Special attention has been given to TRPC’s role in cardiac hypertrophy, but the implication of TRPV1, TRPV2, and TRPM4 has been also demonstrated.

#### TRPCs and Hypertrophy

The role of TRPCs in the pathological cardiac hypertrophy has been extensively studied in isolated neonatal and adult cardiac myocytes and in different animal model of cardiac hypertrophy (Nishida and Kurose, 2008; Watanabe et al., 2008; Eder and Molkentin, 2011; Xiao et al., 2017). Ca^2+^ entry through TRPCs is considered essential to activate signaling pathways involving PKC, calmodulin-kinase, and calcineurin/NFAT, which promotes cardiac hypertrophy by the re-expression of the fetal gene program (Heineke and Molkentin, 2006; Nakayama et al., 2006; Eder and Molkentin, 2011). Independent reports demonstrated that stimulation of neonatal rat cardiac myocytes with hypertrophic agonists such as ATP, endothelin-1, phenylephrine, or angiotensin-II, increased cells size, correlating with the upregulation and activation of TRPC1 (Ohba et al., 2007), TRPC3 (Brenner and Dolmetsch, 2007), TRPC5 (Sunggip et al., 2018), and TRPC7 (Satoh et al., 2007). Using animal models of pressure overload-induced heart hypertrophy, different studies demonstrated that TRPC1 (Ohba et al., 2007), TRPC3 (Bush et al., 2006; Brenner and Dolmetsch, 2007), and TRPC6 (Kuwahara et al., 2006) are upregulated in heart. Conversely, the overexpression of TRPC3 in transgenic mice increased cardiac hypertrophy through calcineurin/NFAT activation when mice were subjected to angiotensin-II and phenylephrine treatment or pressure overload (Nakayama et al., 2006). Similarly, the overexpression of TRPC6 in transgenic mice leaded to the development of cardiac hypertrophy and heart failure (Kuwahara et al., 2006). In contrast, the use of cardiomyocyte-specific dominant-negative mutants in transgenic mice for TRPC3 and TRPC6 or for TRPC4 reduced cardiac hypertrophic responses following either the infusion of neuroendocrine agonists or pressure overload stimulation through calcineurin/NFAT (Wu et al., 2010). Moreover, *trpc1* gene deletion in mice attenuated pressure overload-induced hypertrophy by the alteration of calcineurin/NFAT and Akt signaling pathway (Seth et al., 2009). Intriguingly and in contrast to other studies mentioned here, a previous study reported that TRPC3/TRPC6 double knockout mice did not develop pressure-overload induced hypertrophy; however, *trpc3* or *trpc6* gene’s deletion did not protect the heart from hypertrophy or dysfunction due to pressure overload, suggesting the need of both channels to promote the deleterious hypertrophic response (Seo et al., 2014). This finding worth further investigation and confirmation in other model of pathological cardiac hypertrophy.

#### TRPVs and Hypertrophy

Most studies focused on TRPV1 and TRPV2 role in hypertrophy and the adverse cardiac remodeling. An early study revealed that TRPV1 knockout mice showed a reduced increase in heart’s weight as compared to wild-type mice, and they were partially protected from pressure-overload induced cardiac hypertrophy (Buckley and Stokes, 2011). A recent study demonstrated that TRPV1’s expression is increased in hypertrophied heart of mice evoked by transverse aorta constriction (TAC) (Chen et al., 2016). However, *Trpv1* gene’s deletion was associated with excessive inflammation, exaggerated cardiac hypertrophy, and abnormal cardiac function after TAC, suggesting a protective role of TRPV1 (Zhong et al., 2018a). This study proposed that TRPV1, highly expressed in sensory nerves, might be involved in the regulation of cardiovascular function due to its anti-inflammatory effects *via* calcitonin gene-related peptide. Conversely, a previous study revealed that pharmacological activation of TRPV1 with SA13353 prevents the increase of cardiomyocyte size evoked by endothelin-1 (Zhang et al., 2012b). SA13353 also attenuated and reduced cold stress-induced hypertrophy and decreased the fractional shortening among others functional cardiac and cellular parameters (Zhang et al., 2012b). In contrast, another study showed that TRPV1 activation with capsaicin can antagonize high-salt diet-mediated cardiac hypertrophy, by ameliorating the mitochondrial complex I oxidative phosphorylation and suggesting that TRPV1-mediated amendment of mitochondrial dysfunction may represent a novel target for the management of early cardiac dysfunction (Lang et al., 2015). Therefore, the role of TRPV1 in the molecular mechanism underlying pathological cardiac hypertrophy still remains unclear.

TRPV2 is also significantly upregulated in wild-type mice subjected to TAC, whereas the absence of functional TRPV2 reduces significantly the left ventricular hypertrophy after TAC, suggesting a role of TRPV2 in the development of cardiomyocyte hypertrophy because of an increased afterload (Koch et al., 2017). In this way, the overexpression of TRPV2 was also associated with enlarged hearts in patients with dilated cardiomyopathy (Iwata et al., 2013). TRPV3 is also overexpressed in angiotensin-II-induced cardiomyocyte hypertrophy, which aggravated cardiac hypertrophy through calcineurin/NFATc3 signaling pathway activation (Zhang et al., 2018).

#### TRPMs and Hypertrophy

In the case of TRPMs, different studies focused especially on TRPM4 role in hypertrophy, while little is known regarding other TRPM channels. TRPM4 is thought to fine-tune the amount of Ca^2+^ influx into cardiomyocytes via store-operated Ca^2+^ channels after chronic angiotensin-II stimulation, through a mechanism involving calcineurin–NFAT activation (Kecskés et al., 2015). TRPM4 upregulation was observed in hypertrophied ventricular cardiomyocytes freshly isolated from well-established genetic model of spontaneously hypertensive rats when compared to control, the Wistar Kyoto rats (Guinamard et al., 2006). Interestingly, selective removal of TRPM4 from the heart resulted in an increased hypertrophic growth after chronic mice treatment with angiotensin-II as compared to wild type mice (Kecskés et al., 2015), suggesting a protective role of TRPM4. Recently, a study confirmed the beneficial role of TRPM4 in hard training-induced physiological hypertrophy because TRPM4 knockout mice developed a pathological cardiac hypertrophy when they were subjected to endurance training (Demion et al., 2014). The deletion of the *Trpm4* gene in 12-week-old mice was linked with moderate cardiac hypertrophy as well as ventricular dilation, increased cellular density, and reduced left ventricular cardiomyocytes size, suggesting that TRPM4 might act as a negative regulator of cardiomyocytes proliferation during prenatal development (Demion et al., 2014).

### Role of TRPs in Interstitial Fibrosis

**Figure 2:**
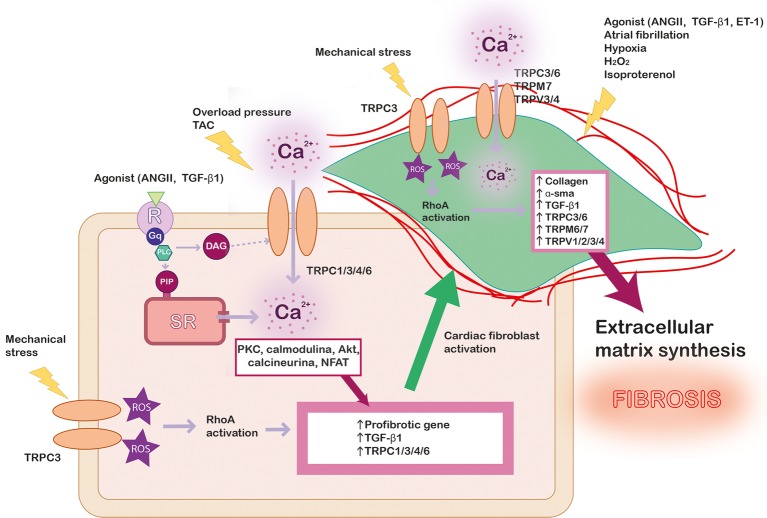
Scheme summarizing the role of TRP channels in cardiac fibrosis. In pathological conditions, different kind of stress stimulates Ca^2+^ entry in cardiac myocyte through TRP channels and other signaling pathway as RhoA dependent on reactive oxygen species (ROS) production, which lead to profibrotic gene’s expression. Profibrotic agonists and other stimuli activate cardiac fibroblast (green) leading to their proliferation and differentiation. The intracellular Ca^2+^ concentration increase through TRP channels promotes the expression of pro-fibrotic agonist (TGF-β1), α-SMA, collagen, and different isoforms of TRP channels, leading to exacerbated extracellular matrix synthesis and fibrosis.

Cardiac fibroblasts represent ∼75% of all cardiac cells, although they represent about ∼10–15% of total cardiac cell volume due to their small size. However, the activation of cardiac fibroblasts plays a crucial role in cardiac pathology. Cardiac fibrosis is caused by an excessive extracellular matrix proteins produced by myofibroblasts, the differentiated phenotype of fibroblasts under stress situations (Yue et al., 2013), where the Ca^2+^ signaling is essential for transcriptional control regulation and myocardial fibrosis (Ramires et al., 1998), as illustrated in Figure 2. The role of TRP channels in cardiac fibroblasts activation, proliferation, and differentiation has been extensively investigated (Du et al., 2010; Yue et al., 2013; Certal et al., 2017). However, unlike in cardiac myocytes, TRPs-activated Ca^2+^ signaling mechanisms are not fully understood in fibroblasts.

#### TRPCs and Fibrosis

TRPC1, TRPC3, and TRPC6 are considered the main TRPCs implicated in cardiac fibrosis, but most studies focused on TRPC3’s role in cardiac fibrosis, as reviewed recently (Numaga-Tomita et al., 2017). A role of background Ca^2+^ entry through TRPC1 and TRPC4 was associated with fibrosis in double knockout mice subjected to pressure overload (Camacho Londoño et al., 2015). In mechanically stressed hearts, TRPC3 activation triggered an aberrant increase of ROS production, leading to RhoA activation in both cardiac myocytes and fibroblasts, resulting in interstitial fibrosis (Kitajima et al., 2016). Actually, *trpc*3 gene deletion suppressed cardiac fibrosis in response to pressure overload or to angiotensin-II infusion (Domes et al., 2015; Kitajima et al., 2016; Numaga-Tomita et al., 2016). TRPC3 blockade with Pyr3 also inhibited angiotensin-II-induced Ca^2+^ entry, proliferation, and differentiation of fibroblasts isolated from left atrial of electrically maintained atrial fibrillation in a dog model (Harada et al., 2012). Recently, chronic treatment of rat models of pressure overload with GSK503A, proposed to inhibit both TRPC3 and TRPC6, showed no interstitial cardiac fibrosis (Seo et al., 2014), suggesting that TRPC3 and TRPC6 are needed for the fibrosis appearance. TRPC6 has been supposed as a regulator of myofibroblast differentiation, since its silencing in human cardiac fibroblasts attenuated the upregulation of α-SMA, a marker of myofibroblast transformation, induced by TGF-β1, a pro-fibrotic agonist (Kapur et al., 2014). Conversely, the loss of TRPC6 in knockout mice prevented TGF-β1-mediated myofibroblast transformation. In addition, TRPC6 overexpression using adenovirus promoted fibroblast conversion to myofibroblast, a hallmark of fibrosis (Davis et al., 2012). In contrast to this finding, an early report proposed that silencing of TRPC6 enhanced myofibroblast formation (Nishida et al., 2007), which questions whether TRPC6 is relevant for cardiac fibrosis.

#### TRPVs and Fibrosis

TRPV channels seem also critical for cardiac fibroblast differentiation to myofibroblasts. It was shown that the ablation of TRPV1 markedly enhanced the fibrosis due to myocardial infarction, by the stimulation of the TGF-β1 and Smad2 signaling pathway (Huang et al., 2010). In contrast, the administration of a TRPV1 antagonist in TAC mice protected the heart from the fibrotic process (Horton et al., 2013). Conversely, in a pressure-overload mice model, it demonstrated a reduction of hypertrophy and fibrosis-mediated TRPV1 channel activation by capsaicin (Wang et al., 2014). Capsaicin blunted pressure overload–induced upregulation of TGF-β1 and Smad2/3 phosphorylation. It also reduced angiotensin-II-induced proliferation of cardiac fibroblast isolated from wild-type but not from TRPV1-knockout mice (Wang et al., 2014). Recently, the overexpression of TRPV1 in transgenic mice-attenuated isoproterenol-induced myocardial fibrosis *in vitro* and *in vivo* in primary cultured cardiac fibroblasts (Wang et al., 2016).

Other reports suggested that TRPV2, TRPV3, and TRPV4 channels also participate in cardiac fibrosis. TRPV2 functional deletion was associated with a decreased development of fibrosis associated with aging (Jones et al., 2017), while TRPV2 upregulation was associated with enlarged hearts, increased fibrosis, and myocardial structural defects in patients with dilated cardiomyopathy (Iwata et al., 2013). Furthermore, TRPV3 activation intensified cardiac fibrosis, stimulating cardiac fibroblast proliferation in the pressure-overloaded rat hearts (Liu et al., 2018). TRPV4 seems also necessary for cardiac fibroblasts differentiation to myofibroblasts, since AB159908, a TRPV4 antagonist, as well as TRPV4 siRNA inhibited TGF-β1-induced fibroblasts differentiation (Adapala et al., 2013). Actually, TRPV4-knockout mice presented decreased fibrosis after myocardial infarction (Adapala et al., 2018).

#### TRPMs and Fibrosis

Several reports made special attention to the abnormal expression of TRPM7 and the development of cardiac fibrosis; however, little is known regarding other TRPM channels (Xu et al., 2015). Recently, higher amount of mRNA and protein levels of TRPM6 in addition to significant increase in markers of myocardial fibrosis as TGF-β1, collagen I and III, were detected in atrial fibrillation patients, suggesting possible contribution of TRPM6 to atrial fibrosis (Zhang et al., 2015).

Experimental strategies treating human or animal cardiac fibroblasts with pro-fibrotic agonists or with hormones as angiotensin-II increased significantly the expression of TRPM7. In fact, TGF-β1 addition to human atrial fibroblasts upregulated the expression of TRPM7; meanwhile, TRPM7 silencing inhibited fibroblasts proliferation, differentiation, and collagen production induced by TGF-β1 (Du et al., 2010). Angiotensin-II also stimulated TRPM7 resulting in an increased expression of α-SMA and fibronectin protein (Yu et al., 2014). Moreover, rat cardiac fibroblasts incubated with angiotensin-II increased levels of protein expression of TRPM7, collagens I and III, which promoted fibrosis (Zhou et al., 2015). In contrast, the downregulation of TRPM7 decreased its related current density and inhibited angiotensin-II mediated cardiac fibroblasts proliferation, differentiation, and collagen synthesis (Li et al., 2017). Moreover, in rats with sick sinus syndrome, TRPM7 regulated angiotensin II-induced cardiac fibroblasts proliferation and collagen synthesis of sinoatrial node, involving Smad signaling pathway (Zhong et al., 2018b). Recently, a role of TRPM7 was reported in miRNA-135a inhibition of isoproterenol-induced cardiac fibrosis (Wu et al., 2018) and in fibrosis evoked by H_2_O_2_ and hypoxia (Guo et al., 2014; Lu et al., 2017). Therefore, TRPM7 stand out as an interesting possible target to attenuate pathological cardiac fibrosis.

### TRPs and Conduction Disorders

Atrial fibrillation (AF) is the most sustained clinical arrhythmia, which occurs due to structural remodeling, involving prominent fibrotic changes (Yue et al., 2011). TRP channels do not influence the excitability of heart’s pacemaker but apparently their upregulation mediate the arrhythmogenesis and the progression of electrical remodeling of the diseased heart (Yue et al., 2015). Special advances have been made by studying the role of TRPM4, TRPC3, and TRPM7 in conduction disorders in the heart.

TRPM4 contribution to cardiac conduction as well as the development of arrhythmias has been demonstrated using different approaches, such as channel inhibition with 9-phenantrol, a blocker for TRPM4 (Grand et al., 2008), using TRPM4-deficient mouse models, and by the identification of TRPM4 mutants detected in a variety of inherited human cardiac arrhythmias (Freichel et al., 2017). A pro-arrhythmic role of TRPM4 and its participation in membrane potential depolarization likely explain the triggering of spontaneous beating and the increase of action potential duration described in the hypertrophied heart (Demion et al., 2014). *In vitro* experiments showed that 9-phenanthrol decreased Ca^2+^ oscillations in atrial HL-1 mouse cardiac myocytes, thought to play a critical role in arrhythmias (Burt et al., 2013). TRPM4 inhibition with 9-phenanthrol also mimicked the reduction of action potential duration evoked by TRPM4 deletion in atrial cells (Simard et al., 2013) and reverted the early after-depolarization involved in cardiac arrhythmias observed after a process of hypoxia and re-oxygenation (Simard et al., 2012). Recently, it was shown that physiological range of [Ca^2+^]_i_ could activate TRPM4, and its upregulation altered action potential characteristics in HL-1 cells treated with angiotensin-II, which increased the arrhythmic propensity of cardiac tissue in pathological situation (Hu et al., 2017). Interestingly, *trpm4* gene mutations were linked to progressive familial heart block type 1 (Kruse et al., 2009; Daumy et al., 2016), isolated cardiac conduction disease (Liu et al., 2010), atrio-ventricular block (Stallmeyer et al., 2012; Syam et al., 2016), right bundle branch block (Stallmeyer et al., 2012), Brugada syndrome (Liu et al., 2013; Gualandi et al., 2017), and recently to either complete heart block or idiopathic ventricular fibrillation (Bianchi et al., 2018).

Additional indications suggested that TRPM7 and TRPC3 might also mediate the pathogenesis of AF. In atrial fibroblasts from AF patients, TRPM7 is notably upregulated (Du et al., 2010; Zhang et al., 2012a) and was suggested to play a pivotal role in AF due to fibrogenesis in the atrium since fibrosis is the main factor for AF. TRPM7 knockdown suppressed endogenous TRPM7 currents, decreased Ca^2+^ influx in atrial fibroblasts, and inhibited TGF-β1-induced fibroblast proliferation, differentiation, and collagen production (Du et al., 2010). TRPC3 is also significantly upregulated in the atria of AF patients (Zhao et al., 2013). In fibroblasts freshly isolated from left atrial of dogs undergoing AF, by sustained atrial tachypacing, it was observed a significantly increase in TRPC3 protein expression, currents, ERK phosphorylation, and extracellular matrix gene expression (Harada et al., 2012). Further evidence for a role of TRPC3 has been demonstrated in experiments using TRPC3 knockout mice, in which the effect of angiotensin-II addition to pacing-induced AF mice was significantly reduced compared to wild-type mice (Ju et al., 2015). Interestingly, a recent study examined the expression of different TRPs in leukocytes of patients with nonvalvular AF (Düzen et al., 2017). They observed marked increase in gene expression of TRPC1-C7, TRPV1-V6, TRPM1-M8, TRPML1-ML3, TRPA1, and TRPP2. A possible correlation between these leukocytes genes’ expression and those examined from the atrium will be of major interest. Therefore, further investigations are undoubtedly needed for understanding the role of all these TRP channels in AF.

## Conclusions and Perspectives

A growing body of evidence has demonstrated that, by controlling Ca^2+^ homeostasis, different TRP isoforms are critically involved in pathological cardiac remodeling and heart failure. However, molecular mechanisms which trigger the transition of the heart from adaptation to maladaptation by these channels are still unknown. In the last two decades, the use of genetically modified animal and mice models of cardiac disease provided valuable information about TRPs implication in cardiac remodeling. Nevertheless, substantial work is still required to understand why many TRPs from different subfamilies are activated by similar pro-hypertrophic or pro-fibrotic stimuli, and whether they associate or interact between them to activate signaling pathway involved in hypertrophy, fibrosis, or conduction disorders. Unfortunately, the wide range of agonists and antagonists used to modulate TRPs failed to determine which TRPs might be the right target(s) to characterize and consider as therapeutic tools. More specific inhibitors/activators of TRPs are eagerly awaited to shed a light on the complex mechanism of cardiac diseases associated with remodeling. Of hope, the increasing amount of available information related to TRP-drug interaction sites and gating processes of TRP channels is expected to facilitate the development of novel therapeutic concepts by pharmaceutical companies. Overall, and in the light of the reported studies, TRP channels are still considered promising therapeutic targets to regulate pathological cardiac plasticity.

## Author Contributions

TS conceived the concept of the review. TS, IG-O, DF, RT, MM-B, and EC-S wrote the review. IG-O, DF, AH and JR designed and formatted the figures. TS, IG-O, RT, DF, AH, and JR read and edited the review manuscript.

### Conflict of Interest Statement

The authors declare that the research was conducted in the absence of any commercial or financial relationships that could be construed as a potential conflict of interest.

## Abbreviations

AF: Atrial Fibrillation
[Ca^2+^]i: concentration of intracellular Ca^2+^
ROS: reactive oxygen species
SOCE: store-operated Ca^2+^ entry
TRP: transient receptor potential
TRPC: transient receptor potential-canonical
TRPV: transient receptor potential-vanilloid
TRPM: transient receptor potential-melastatin
TRPA: transient receptor potential-ankyrin
TRPML: transient receptor potential-mucolipin
TRPP: transient receptor potential-polycystin.
